# Partial Purine Nucleoside Phosphorylase Deficiency: an Unexpected Diagnosis in an Adult Patient

**DOI:** 10.1007/s10875-025-01941-8

**Published:** 2026-02-02

**Authors:** Manisha Ahuja, Lynette Fairbanks, Catherine Stroud, Andrew Schaefer, Suzanne Elizabeth Elcombe

**Affiliations:** 1 Specialist Registrar in Immunology, Royal Victoria Infirmary, Newcastle upon Tyne Hospitals NHS Foundation Trust, Newcastle upon Tyne, United Kingdom; 2 Clinical Scientist at Viapath, Purine Research Laboratory, St Thomas’ Hospital, London, United Kingdom; 3 Consultant Neurologist, Royal Victoria Infirmary, Newcastle upon Tyne Hospitals NHS Foundation Trust, Newcastle upon Tyne, United Kingdom; 4 Consultant Immunologist, Royal Victoria Infirmary, Newcastle upon Tyne Hospitals NHS Foundation Trust, Newcastle upon Tyne, United Kingdom

To the Editor,

## Introduction

Purine Nucleoside Phosphorylase (PNP) is a ubiquitous enzyme found in the purine degradation and salvage pathways. It metabolises inosine and deoxyinosine into hypoxanthine, and guanosine and deoxyguanosine into guanine. PNP deficiency leads to accumulation of deoxyguanosine triphosphate (dGTP) which inhibits DNA synthesis and repair in affected cells. This enhances apoptosis in rapidly replicating or those exposed to oxidative stress, typically resulting in immunotoxicity and lymphopaenia.

PNP deficiency is caused by biallelic mutations in the *PNP* gene located at chromosome 14q13.1. It classically presents in the early years of life with a combined immunodeficiency and often autoimmunity and haematological malignancies. Patients typically have lymphopaenia, reduced serum urate, poor T cell proliferation, absent vaccine specific antibodies, reduced or absent PNP enzymatic activity in red blood cells and excess urinary and blood purine substrates (inosine, deoxyinosine, guanosine, and deoxyguanosine).

Neurological symptoms (ataxia, hypotonia, developmental delay, and spasticity) are seen in two thirds of patients however the mechanism behind these defects is unknown. Accumulation of dGTP cannot fully explain the range of neurological phenotype. One proposed mechanism is reduced availability of GTP, an essential neurotransmitter in the central nervous system (CNS) due to PNP deficiency. In some patients who have had curative haematopoietic stem cell transplantation (HSCT), neurological damage has often arrested (but not improved), with some other cases showing improvement with development of new neurological milestones. Studies in mice show that early treatment with enzyme replacement can prevent cerebellar damage supporting an early diagnosis for prevention of neurological sequelae [1].

Grunebaum et al. [2] to date have described the oldest individuals diagnosed with PNP deficiency; three siblings with a homozygous mutation(c.769 C > G;p.His257Asp) in the third decade of their lives. The 21 year old female proband had recurrent sino-pulmonary infections, one of two male siblings (aged 25) was unaffected and the other (aged 28) had childhood infections and fine motor issues that subsequently resolved. PNP activity was reduced in two siblings 8–11% of control (third not measured) and all three had increased urinary substrates. The review discussed two previously reported cases. The first is a 13 year old patient with late onset PNP deficiency who developed recurrent infections at the age of 6 and successfully underwent HSCT, aged 14. The second, a 21 year-old pregnant female with absent PNP who remained largely infection-free since her presentation at 3 years of age. She was treated with prophylactic antibiotics and immunoglobulin.

In this letter, we describe a patient diagnosed with PNP deficiency at the age of 50, which we understand to be the oldest known diagnosis of this condition.

### Presentation

Our patient was delivered very floppy at full term. Delayed milestones were noted and she started walking by age three. She had difficulties reading and writing, developed action induced dystonia, myoclonus, chorea, and a symptomatic macular dystrophy. She had worsening mobility over her teenage years requiring use of a frame aged 13. By the age of 23, she was only able to mobilise with a wheelchair due to generalised dystonia. Reflexes were brisk, but there was no convincing spasticity or ataxia. MRI brain was normal and DAT scan was consistent with nigrostriatal dopaminergic deficiency.

In her late teenage years, she had recurrent viral and plain warts on her trunk, limbs and hands. She required multiple surgeries and liquid nitrogen therapy. Aged 24, she developed a progressive rash on her left foot and by age 37 had significant dry, erythematous and hardened plaques on her left calf. Biopsies showed skin granulomas. She reported recurrent chest and sinus infections in her thirties and required admission to hospital on three occasions with pneumonia. High Resolution CT Chest confirmed bronchiectasis. She was started on azithromycin prophylaxis and in 2009, aged 39, she was referred to immunology for further assessment.

### Investigations

Immunological investigations showed absent vaccine specific responses, T and B cell lymphopaenia and absent memory B cells. Immunoglobulin replacement commenced in 2009 was successful in reducing infection frequency. In 2019, aged 50, she participated in the UK 100,000 genomes project for diagnosis of rare diseases. A homozygous variant {NM_000270.3(PNP): c.701G > C(Arg234Pro)} was identified in DNA from whole blood. This has previously been reported in five unrelated individuals [3] that were compound heterozygous with another pathogenic variant. Aust et al. [4], transfected a COS cell line with this allele and noted absence of any human PNP enzymatic activity in cell lysates.

We undertook further analysis to investigate PNP enzyme activity in patient cells at the Purine Laboratory in St Thomas’ Hospital, London. Whole blood was separated into red blood cells (RBCs), white blood cells (WBCs) and platelet lysates. PNP activity was measured using HPLC and inosine as substrate. Minimal PNP concentration was noted in RBCs (1nmol/h/mg; normal range 3000–7000; carrier 100–3000; deficient < 100), WBCs (431nmol/h/mg; mean for controls 20566nmol/h/mg) as well as platelets. Surprisingly, purine nucleosides in urine (inosine, guanosine, deoxyinosine and deoxyguanosine) were completely absent.

Our variant causes a switch from arginine to proline close to the binding pocket for metabolites in the enzymatic structure. We hypothesised that this may lead to enzyme fragility and the process of creating lysates may artificially disrupt this fragile enzyme. We therefore undertook testing in intact RBCs incubated with inosine, with levels of both inosine and hypoxanthine measured over time. RBCs incubated with Earle’s balanced salt solution (EBSS) were used as negative control. This assay (Table 1) shows increasing amounts of hypoxanthine, and decreasing amounts of inosine over time in our test sample, implying metabolism of inosine to hypoxanthine by PNP. PNP activity calculated in intact RBCs was now close to the carrier status at 98 nmol/h/mg. The negative control showed no change in hypoxanthine concentration.

**Table 1:** Analysis of PNP activity in intact rbcs: levels of hypoxanthine and inosine in patient samples when mixed with and without inosine

	Patient RBCs mixed with inosine and EBSS		Patient RBCs mixed with EBSS only
Time (minutes)	Hypoxanthine (umol/l)	Ionisine (umol/l)	Hypoxanthine (umol/l)
5	130.9	912	6
10	201.3	818.3	7
30	314.7	580.9	7.4

To investigate the possibility of somatic mosaicism, we obtained a dermal biopsy and generated fibroblasts for investigation of PNP activity in non-haematopoietic cells. Patient fibroblasts showed reduced, but crucially, present PNP activity in the patient samples (295nmol/h/mg) compared with control 1 (1340nmol/h/mg) and control 2 (1232nmol/h/mg). Previously established range for this assay using 19 controls is between 562–4256nmol/h/mg.

## Discussion

To our knowledge, we describe the oldest diagnosis of partial PNP deficiency to date. Despite the significant neurological symptoms, our patient’s immunological phenotype is mild compared to reported classical cases of complete PNP deficiency. In contrast to the in vitro work undertaken by Aust et al. using lysates [4], our intact RBC incubations show partial PNP activity more in keeping with a carrier status. The lack of urinary substrates is surprising, but supports the hypothesis that accumulation of dGTP is not the only mechanism of action for neurological deficit in these patients.

Interestingly, there are reported cases where patients presenting with SCID due to ADA deficiency, (an enzyme required for purine metabolism upstream of the PNP enzyme) have spontaneously improved with partial clinical remission due to monoallelic reversion of the pathogenic mutation leading to a state of somatic mosaicism in the patients [5]. The possibility of a somatic mosaicism in this case could be further investigated by genotyping different patient tissues such as fibroblasts. Further functional studies assessing the enzymatic stability in this variant and its impact on the immune and central nervous systems would be also very enlightening.

## References

[1] Karaaslan G, et al. Neurologic status of patients with purine nucleoside phosphorylase deficiency before and after hematopoetic stem cell transplantation. J Clin Immunol. 2023;43:1. 10.1007/s10875-023-01585-6. 37726596 10.1007/s10875-023-01585-6

[2] Grunebaum E, et al. Partial purine nucleoside phosphorylase deficiency helps determine minimal activity required for immune and neurological development. Front Immunol. 2020;11:1257. 32695102 10.3389/fimmu.2020.01257 PMC7338719

[3] Walker PL, et al. Purine nucleoside phosphorylase deficiency: a mutation update. Nucleosides Nucleotides Nucleic Acids. 2011;30(12):1243–7. 22132981 10.1080/15257770.2011.630852

[4] Aust MR, et al. Molecular analysis of mutations in a patient with purine nucleoside phosphorylase deficiency. Am J Hum Genet. 1992;51(4):763–72. 1384322 PMC1682776

[5] Moncada-Vélez M, et al. Somatic mosaicism caused by monoallelic reversion of a mutation in T cells of a patient with ADA-SCID and the effects of enzyme replacement therapy on the revertant phenotype. Scand J Immunol. 2011;74:471–81. 21671975 10.1111/j.1365-3083.2011.02593.x PMC3188688

